# Insight into Generation and Evolution of Sea-Salt Aerosols from Field Measurements in Diversified Marine and Coastal Atmospheres

**DOI:** 10.1038/srep41260

**Published:** 2017-01-25

**Authors:** Limin Feng, Hengqing Shen, Yujiao Zhu, Huiwang Gao, Xiaohong Yao

**Affiliations:** 1 Key Lab of Marine Environmental Science and Ecology, Ministry of Education, Ocean University of China, Qingdao, China; 2 State Key Laboratory of Environmental Simulation and Pollution Control, College of Environmental Sciences and Engineering, Peking University, Beijing 100871, China; 3 Qingdao Collaborative Center of Marine Science and Technology, Qingdao 266100, China

## Abstract

This report focuses on studying generation and/or evolution of sea-salt aerosols (SSA) on basis of measurements in the Northwest Pacific Ocean (NWPO), the marginal seas of China, at sea-beach sites and a semi-urban coastal site in 2012–2015. From measurements in the NWPO, we obtained the smallest generation function of the super-micron SSA mass ([M_SSA_]) by the local wind comparing to those previously reported. Vessel-caused wave-breaking was found to greatly enhance generation of SSA and increase [M_SSA_], which was subject to non-natural generation of SSA. However, naturally enhanced generation of SSA was indeed observed in the marginal seas and at the sea-beach site. The two enhancement mechanisms may explain the difference among this and previous studies. Size distributions of super-micron SSA exhibited two modes, i.e., 1–2 μm mode and ~5 μm mode. The 1–2 μm mode of SSA was enhanced more and comparable to the ~5 μm mode under the wind speed >7 m/s. However, the smaller mode SSA was largely reduced from open oceans to sea-beach sites with reducing wind speed. The two super-micron modes were comparable again at a semi-urban coastal site, suggesting that the smaller super-micron mode SSA may play more important roles in atmospheres.

Sea-salt aerosols (SSA) are the suspension produced directly at the sea surface. SSA are ones of the main sources of particulate matter in marine and coastal atmospheres and play an important role in chemical conversion of reactive compounds therein and the global radiation balance^1^,^2^,^3^. NaCl is the most main component of super-micron (>1 μm) SSA, but its contribution to the mass of sea spray aerosols is largely reduced in the sub-micron (0.1–1 μm) size range^4^. In the atmosphere, SSA can rapidly age through a series of physical and chemical processes including heterogeneous reactions, cloud and fog processes^5^,^6^,^7^,^8^,^9^,^10^. For example, alkaline surface of SSA was reported to provide a perfect place for atmospheric chemistry reactions under ambient conditions rich in acid gases^8^,^11^,^12^. The chemical processes deeply affect nitrogen and chloride cyclings in marine atmospheres^1^,^13^,^14^,^15^.

Numerous laboratory experiments have been devoted to identify three types of directly released drops at the sea surface, which are conventionally referred as film drops (fragments of the collapsed bubble cap), jet drops (shot from the cavity formed after the bubble bursting), and spume drops (generated from wave tearing by sufficient high wind stress)^16^,^17^. The film drops are evaporated to generate sub-micron atmospheric aerosols mainly consisting of organic matter while the latter two drops are generally evolved to generate super-micron atmospheric aerosols overwhelmingly consisting of sea-salt^18^,^19^,^20^,^21^,^22^. However, the characteristics of SSA evolved from daughter droplets of jet drops and spume drops remain poorly understood^23^. Recently, de Leeuw *et al*.^24^ reviewed the laboratory studies of bubble-bursting droplets and concluded that the studies previously reported in literature, typically bubbled in tanks or other generators, were not performed under strict open ocean conditions^24^.

The mass concentration of SSA (M_SSA_) measured in open oceans was reported to depend mainly on the local surface wind stress^25^. There are several processes responsible for the removal of SSA, i.e., gravitational sedimentation for larger SSA and precipitation for smaller SSA. Considerable efforts have been devoted to derive SSA production functions. The relevant physical parameters of SSA production functions include the local wind speed and sea surface temperature (SST)^26^,^27^,^28^,^29^. The most concise empirical relationship between local surface wind speed (usually measured at 10 m above the sea level) and the M_SSA_ based on observations is *M*_*SSA*_ = *e*^*aU*+*b*^, in which *a* and *b* are two coefficients and U is the wind speed. Gong *et al*.^27^ reviewed the observations reported in literature and found *a* varying from 0.12 to 0.27^27^. Gong *et al*.^25^ further narrowed down the value of *a* to 0.14–0.17^25^. Wai & Tanner^29^ reported the values of *a* and *b* to be 0.17 and 0.87 at a coastal site in Hong Kong, while Lovett^28^ reported the values of 0.16 and 1.45 in the Atlantic Ocean based on ship measurements at 5–15 m above the sea level^28^,^29^. The causes for varying *a* and *b* remain unclear.

In this study, we launched several field campaigns to measure mass or volume size distributions of SSA in the Northwest Pacific Ocean (NWPO), the Yellow Sea (YS) and the Bohai Sea (BS), at sea-beach sites, and a semi-urban coastal site of Qingdao during different periods in 2012–2015 (Details can be found in *Methods* section). Most parts of the NWPO are subject to oligotrophic regions. The YS and BS are marginal seas and suffer from eutrophication to some extent. Eutrophication is more severe inshore seawaters of the marginal seas because of the rapid development of mariculture industry and sewage discharge. The observations and analysis results in the diversified coastal and marine atmospheres provide new insights on generation and evolution of different-sized SSA.

## Results and Discussion

### M_SSA_ observed in marine and coastal atmospheres

**Figure 1:**
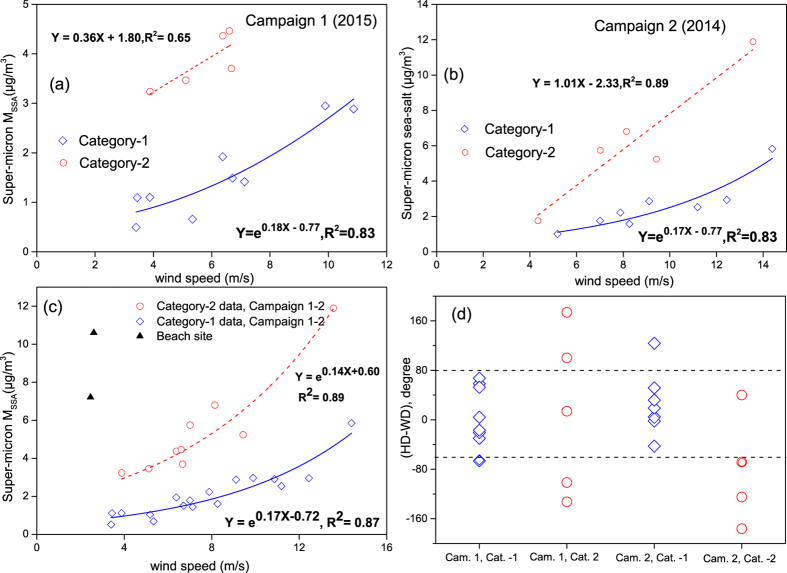
The relationship between super-micron M_SSA_ and wind speed, (**a**,**b**): the estimated M_SSA_ values in super-micron particles against the wind speed in the Campaign 1–2 (the NWPO campaign, including roundtrip samples); (**c**) the estimated super-micron M_SSA_ in category-2 data of campaign 1–2 against the wind speed; (**d**): wind direction (WD) relative to vessel’s heading direction (HD), i.e., HD-WD.

The estimated super-micron M_SSA_ were 2.5 ± 1.4 μg/m^3^ during Campaign 1 in 2015 and 4.0 ± 2.9 μg/m^3^ 8 during Campaign 2 in 2014, which accounted for 88–92% of the total M_SSA_, i.e., 2.7 ± 1.4 μg/m^3^ during Campaign 1 and 4.5 ± 3.1 μg/m^3^ during Campaign 2. Thus, we focus on the analysis of M_SSA_ in super-micron particles afterwards. Considering the relationship between the generation of SSA and the local wind speed, we plotted the estimated mass of super-micron M_SSA_ (expressed as [M_SSA_], similarly hereinafter) against the measured wind speed in the NWPO (Fig. 1a,b). It was obvious that the super-micron SSA can be classified into two categories with different responses to the measured wind speed. In Category-1, we used an exponential function, i.e., [*M*_*SSA*_] = *e*^*aU*+*b*^, for regression analysis. The values of *a* and *b* in Campaigns 1–2 were 0.17–0.18 and −0.77, respectively, and the two campaign results were highly consistent. All data in Category-1 were thus combined for regression analysis (Fig. 1c). Then we obtained [*M*_*SSA*_] = *e*^0.17*U*−0.72^ and R^2^ = 0.87.

In Category-2, we found a quite stronger response of the estimated [M_SSA_] to the measured wind speed, i.e., [*M*_*SSA*_] = 0.36 × *U* + 1.80, R^2^ = 0.65 during Campaign 1 and [*M*_*SSA*_] = 1.01 × *U* − 2.33, R^2^ = 0.89 during Campaign 2. The two constants in regression equations were totally different between Campaign 1 and Campaign 2, implying that the measured SSA may include those generated under ocean natural conditions plus those generated under non-natural conditions. When all data in Category-2 obtained during the two campaigns were used for regression analysis, we obtained [*M*_*SSA*_] = *e*^0.16*U*+0.38^ and R^2^ = 0.79. The *a* value (0.16) was also close to 0.17-0.18 obtained in Category-1, but the *b* value was increased substantially from −0.72 to 0.38. The vessel sailing can cause a wave breaking and generate drops as shown in Fig. S1. The drops were evaporated to generate SSA, which was subject to the generation of SSA under non-natural conditions. During the two campaigns, the SSA in Category-1 were measured when the angle of the wind direction relative to the vessel’s heading ranged from −70° to 80° (upwind sailing, Fig. 1d). The SSA generated by vessel-caused wave-breaking was less likely gathered by the samplers. The SSA in Category-2 were mostly measured when the angle was out of the range, except two samples collected on 18 March 2014 and 4 May 2015 when the angle highly varied in the whole sampling duration. When the two samples were removed for regression analysis, we obtained [*M*_*SSA*_] = *e*^0.14*U*+0.60^ with R^2^ = 0.89 (Fig. 1c). In Catogrory 1–2, the concentrations of EC were almost below the detection limit in the super-micron particles, indicating that ship-self contamination due to combustion emissions had a negligible influence on the obtained [M_SSA_] (Table S1).

Thus, we proposed that the increased *b* value in Category-2 was probably contributed by the vessel-caused wave-breaking. Theoretically, the additionally generated SSA was likely determined by three factors, i.e., the height of sea wave, the angle of the wind direction relative to vessel’s heading and the relative wind speed between. The additional SSA source could be too small to exert a significant influence on the collected SSA in Category-1, but its contribution apparently double that of ocean-naturally generated SSA in Category-2.

During Campaign 3 and Campaign 4, the estimated [M_SSA_] were and 4.0 ± 3.1 μg/m^3^ in 2012 and 3.8 ± 3.0 μg/m^3^ in 2013. The average values were almost same as the average value estimated in particle samples during Campaign 2. Two Categories were also obtained according to the angle of the wind direction relative to the vessel’s heading, i.e., [*M*_*SSA*_] = *e*^0.16*U*^ with R^2^ = 0.86 (Fig. S2) for Category-1 and only four points subject to Category-2. Based on the new regression equation for Category-1, the response of the obtained [M_SSA_] during Campaigns 3–4 almost doubles that during Campaigns 1–2 at the identical wind speed. This implied that additional generation of SSA by wave-breaking may exist in Category-1 during Campaigns 3–4 cruising over the marginal seas, but the additional wave-breaking generation of SSA was probably subject to the natural generation of SSA in the marginal seas. In addition, even a stronger response of super-micron SSA to wind speed was found at the beach site on 15–16 September 2015 (Fig. 1c). On the two days, the onshore wind was blew from the YS during the most of sampling periods. The estimated [M_SSA_] were as high as 7.2 μg/m^3^ and 10.6 μg/m^3^ on 15 and 16 September and the values were 6–10 times larger than those in Category-1 during Campaigns 1–2 and 3–4 times larger than in Category-1 during Campaigns 3–4 at the same wind speed (WS). It was not surprised that the beach greatly enhanced wave-breaking due to the bottom friction and thereby increased [M_SSA_] accordingly. The enhanced wave-breaking was also subject to one of the natural processes in the sea.

Overall, our calculated *a* values in Category-1 during Campaigns 1–4 were almost same and consistent with those reported in literature. Our calculated *b* values, however, varied between the NWPO and the marginal seas of China and differed from *b* values given by Wai & Tanner^29^ and Lovett^28^. Our calculated *b* values in Category-1 were also smaller than that in Category-2. In addition, the b value at the beach site may be even larger than that in Category-2 on basis of our limited data. All these results pointed to the increased *b* values to be likely caused by enhanced wave-breaking. Only the increased *b* values in Category-1 during Campaigns 3–4 and at the beach site reflected natural enhancement in generating SSA, but not for the increased *b* value in Category-2. The larger *b* values reported in literature may reflect natural enhancement in generating SSA while they may also possibly be subject to the enhanced generation of SSA by non-natural factors like in Category-2.

### Size distributions of Na^+^ and Cl^−^ in atmospheric particles

**Figure 2:**
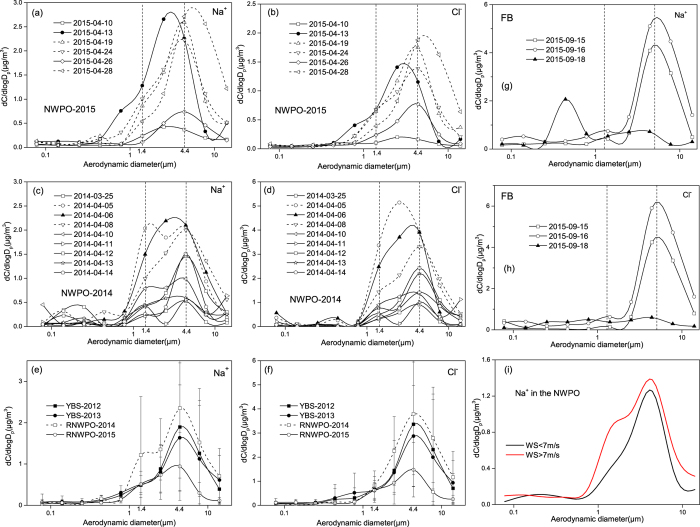
Size distributions of Na^+^ and Cl^−^ during cruise Campaigns 1–4 and beach observations, (**a**,**b**): NWPO-2015 samples; (**c**,**d**): NWPO-2014 samples, the solid symbols represent samples collected during rainfall events, the dash lines for Category-1 samples and the solid lines for category-2 in Fig. 1; (**e**,**f**): the roundtrip samples (open symbols) and the YBS samples (solid symbols); (**g**,**h**): Fushan beach samples, the solid lines for episode samples; (**i**): The mean mass concentrations of Na^+^ divided into two groups in Campaign 1–2 samples using the threshold of WS = 7 m/s. WS represents the wind speed.

During Campaigns 1–2, the samples collected on the way from the south Yellow Sea (SYS) to the NWPO, and the return trip were referred as RNWPO samples and those collected in the NWPO were referred as NWPO samples. When the NWPO samples were considered alone and a multiple log-normal function was used to fit the mass spectra of super-micron SSA, the bi-modal size distributions were obtained for both Na^+^ and Cl^−^ in atmospheric particles, i.e., a mode with the mass median aerodynamic diameter (MMAD) at 1.5 ± 1.6 μm and a mode with MMAD at 5.0 ± 1.7 μm (Fig. 2a–d, Fig. S3). The two modes of Na^+^ were completely isolated in the samples collected on 11 April 2014 with the lowest [M_SSA_] of 1.9 μg/m^3^. The two modes overlapped in some extents in different samples and the 5.0 μm mode usually dominated during Campaign 1 and Campaign 2.

Considering a higher SSA loading associated with a larger wind speed on ocean surface in Campaign 2 (Campaign 1 mean: 6 m/s, Campaign 2 mean: 9 m/s), a larger wind speed likely enhanced the generation of SSA in both super-micron modes. We also found that Na^+^ mass concentrations between two modes correlated significantly (Campaign 1: r = 0.81, P < 0.01; Campaign 2: r = 0.57, P < 0.05 when Pearson correlation was used). A larger wind speed seemingly favors more generation of the smaller super-micron mode Na^+^ than the larger one and the wind speed at 7 m/s was adopted to be the threshold (Fig. 2i). The ratios of Na^+^_1.4μm_/Na^+^_4.4μm_ were 0.32 ± 0.14 under the wind speed <7 m/s for 6 samples and while they were 0.61 ± 0.26 under the wind speed >7 m/s for 9 samples in the NWPO data. SSA generated from film drops mainly exist in sub-micron size. The 5.0 μm mode has been well documented as the generation of SSA from jet drops^24^. Part of jet drops may become unstable and split up into several daughter droplets. The generation of SSA from spume drops was assumed to be important under wind speed >7 m/s^30^. The size of spume drops is commonly believed to be larger than that of jet drops. Evaporation of spume drops likely generated SSA to be mainly distributed at the size range >5.0 μm rather than 1–2 μm. We thus hypothesized the generation of SSA in the 1–2 μm mode from daughter jet drops and/or daughter spume drops. The hypothesis needs to be confirmed by future laboratory experiments.

When size distributions of Na^+^ and Cl^−^ in atmospheric particles in Category-1 (solid lines in Fig. 2) were compared with those in Category-2 (dash lines in Fig. 2), no significant difference existed for the two super-micron mode’s relative abundance between the two categories. The two mode’s relative abundance was apparently determined only by the wind speed regardless of their origins. It should be noted that two samples were collected on rainy days, i.e., on 6 April 2014 and 13 April 2015 when the measured hourly averaged wind speeds arrived at ~11 m/s and 14 m/s, respectively. The major super-micron mode shifted to 4.0 μm on the two day’s samples. The minor super-micron mode slightly shifted to 1.3 μm on 6 April 2014. The direct impacts of rainfall on SSA production are bubble entrainment by the fall of raindrops while the indirect impacts include that production of bubbles by daughter drops generated from raindrops splashing. Both affect the shape of the SSA size distribution^31^. In addition, washout processes may also affect the size distribution of SSA in some extents.

When the RNWPO samples were analyzed (Fig. 2e,f), Category-2 included six out of the total eight samples collected in 2014 and one out of the total four samples collected in 2015. The bi-modal size distributions of Na^+^ and Cl^−^ in super-micron atmospheric particles were observed and the 5.0 μm mode dominated during the RNWPO cruising periods. Again, the two super-micron mode’s relative abundance was likely mainly determined by the local wind speed.

During Campaigns 3–4, the SSA had two super-micron modes with MMAD at 1.5 ± 1.8 μm and 5.0 ± 1.6 μm, consistent with those observed during Campaigns 1–2. The mass ratios of the Na^+^_1.4μm_/Na^+^_4.4μm_ were 0.44 ± 0.31 during Campaign 3 and 0.36 ± 0.21 during Campaign 4, respectively. The mass ratios were smaller than those ratios, i.e., 0.54 ± 0.26 (Category-1 in Campaign 2) and 0.60 ± 0.28 (Category-2 in Campaign 2), although the estimated [M_SSA_] were comparable or even larger. The occurrence frequency of the >7 m/s strong wind was less during Campaigns 3–4 than during Campaigns 1–2, leading to the reduced 1–2 μm mode SSA during Campaigns 3–4.

We further examined size distributions of Na^+^ and Cl^−^ in atmospheric particles collected at a beach site of the YS. The 1.5 ± 1.6 μm super-micron mode was visible for Na^+^ on 15–16 September 2015, but the mass ratios of Na^+^_1.4μm_/Na^+^_4.4μm_ were only 0.11 and 0.15. The ratios were much smaller than those observed in the atmospheres over the NWPO, the YS and BS, although the reverse was true for the [M_SSA_]. The smaller ratios could be due to the sharply reduced generation of SSA from daughter droplets because of no strong wind.

**Figure 3:**
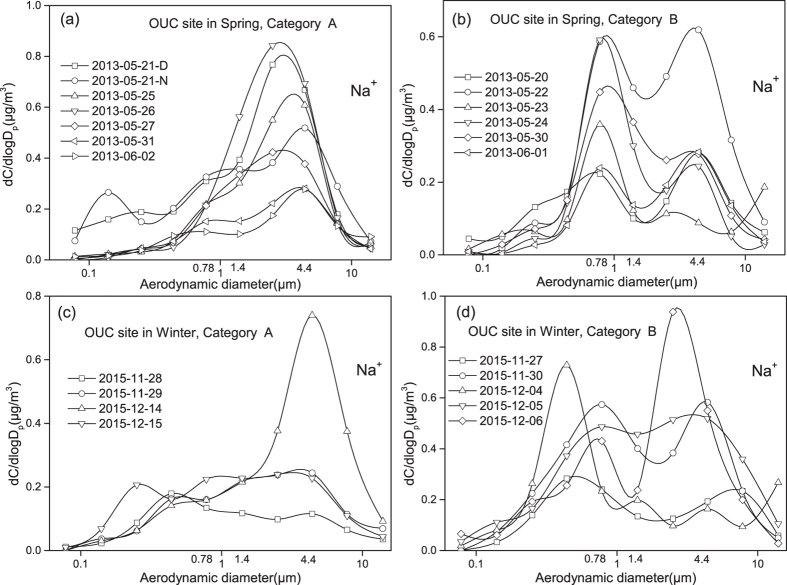
Size distributions of Na^+^ collected at the OUC site in different seasons, (**a**): Category-A data of OUC samples in the spring of 2013; (**b**): Category-B data of OUC samples in the spring of 2013; (**c**): Category-A data of OUC samples in the winter of 2015; (**d**): Category-B data of OUC samples in the winter of 2015.

Figure 3a–d showed the size distributions of Na^+^ in atmospheric particles collected at the semi-urban coastal site in 2013 and 2015. The size distributions can be classified into two categories. Super-micron Na^+^ in Category-A shared similar size distributions with marine samples. In Category-A, the Na^+^ concentration in sub-micron particles was evidently smaller than that of in super-micron particles. This indicated a minor contribution of anthropogenic NaCl to the total NaCl existed in both sub-micron and super-micron atmospheric particles. However, it was not the case for chloride. A strong chloride enrichment existed in sub-micron particles, indicating formation of NH_4_Cl. In Category-A, two super-micron Na^+^ modes can be resolved. For example, the Na^+^ concentration in the 1.5 μm mode particles were sometimes comparable and even larger than that in the 5.0 μm mode particles (Fig. S4). With the distance increasing from the YS, the abundance of SSA at the 1.5 μm mode relative to the 5.0 μm mode likely increased because of the longer residence time for smaller particles. Considered that the site was situated at only ~7 km from the coastline and the averaged wind speed was ~2 m/s during the sampling period, it took approximately one hour for SSA to be transported to the sampling site. The vertical wind speed is usually one order of magnitude smaller than the horizontal wind speed, the abundance of SSA at the 1.5 μm mode relative to the 5.0 μm mode was expected to greatly increase with increasing altitude in the whole troposphere. To better understand the role of 1–2 μm mode SSA in atmospheric chemistry and physics, vertical distributions of SSA need to be studied in various marine atmospheres.

In Category-B, the Na^+^ concentration in sub-micron particles was larger than or close to that in super-micron particles. Sub-micron Na^+^ in Category-B shared similar size distribution with K^+^ and Cl^−^ (Fig. S5), indicating the biomass burning sources. The 1.5 μm mode Na^+^ was thereby unable to be resolved because of the overlap. For these samples, anthropogenic NaCl may play a more important role in atmospheric chemistry and physics than SSA.

### Particle volume concentrations in marine and coastal atmospheres

**Figure 4:**
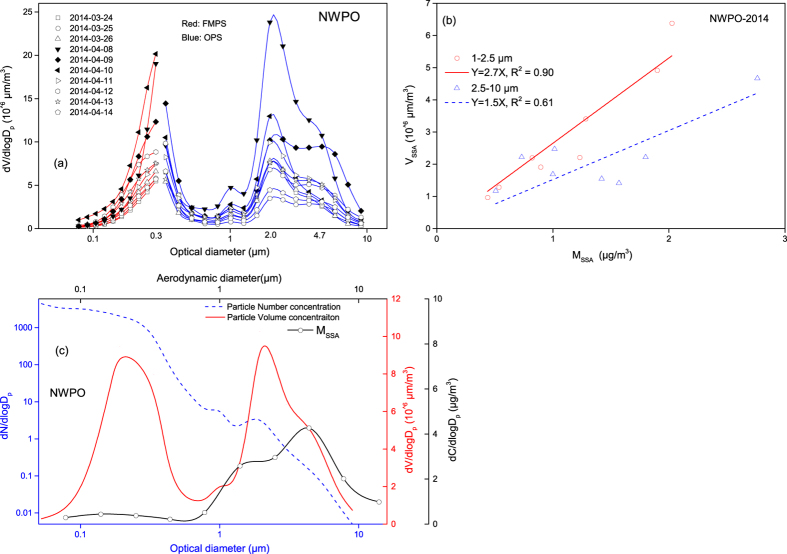
Size distributions of particle volume concentrations in NWPO-2014, (**a**): samples measured by OPS (blue lines) and FMPS (red lines); (**b**): linear regressions between estimated super-micron M_SSA_ and particle volume concentrations of 1–2.5 μm and 2.5–10 μm modes in the NWPO, 2014. (**c**): mean values of estimated M_SSA_, particle number concentrations and particle volume concentration in the NWPO, 2014.

Size distributions of Na^+^ were obtained on basis of time-integrated sampling. Whether two super-micron mode’s SSA simultaneously generated or generated in different times needs to be confirmed by real-time measurements. Particle number concentration data measured by the combined FMPS and OPS were available only in 2014 and Fig. S6 showed size distributions of particle number concentration measured in the NWPO. Assuming atmospheric particles to be spherical, we calculated volume concentrations of different-sized particles shown in Fig. 4a,c. Two super-micron modes of atmospheric particles had the volume median optical diameters (VMOD) 2.1 ± 0.1 μm and 5.1 ± 0.2 μm, respectively, and the two modes were observed in any periods. Since SSA should be the dominant contributor to super-micron particles in the marine atmosphere, the two particle modes most likely correspond to two SSA modes as identified above. We confirmed this by linearly correlating the calculated volume concentrations (V) and the concentrations of Na^+^ in super-micron particles collected at the same sampling periods (Fig. 4b). Good correlations were obtained, i.e., V_1-2.5μm_ = 2.7 × [M_SSA_]_1-2.5μm_, R^2^ = 0.90 and V_2.5-10μm_ = 1.5 × [M_SSA_]_2.5-10μm_, R^2^ = 0.61. The larger determination coefficient for 1–2.5 μm particles than 2.5–10 μm particles can be due to multiple factors such as more chloride in 1–2.5 μm particles depleted by nitric acid as presented later and more non-SSA existed in 1–2.5 μm particles, etc. Moreover, we also calculated the fraction of sodium among all cations, as well as the OMF, in the super-micron particles. Results show that the mass ratios of Na^+^ to the total cation mass in super-micron particles ranged from 0.56 to 0.78 with an averaged value of 0.65. During Campaign 2, sub-micron TOC was detected under the WS >7 m/s. While TOC concentrations in super-micron particles of 3 samples collected on 10–12 April 2014 were above the detection limit, the OMF was only 19%, 13% and 4%, respectively. The results further confirmed the dominant contribution of SSA to the total super-micron particle mass.

Again, the moderately good Pearson correlation (r = 0.75, P < 0.05) was obtained between the two coarse mode volume concentrations. The correlation appeared to strongly rely on three higher concentration samples measured on 8–10 April 2014 when the daily averaged wind speed reached 9–11 m/s. When we ruled out these three samples and the recalculated correlation using the remaining samples, the correlation between 2.1 μm and 5.1 μm modes particles was poor with r = 0.54, P > 0.05. The poor correlation indicated that the two mode SSA were likely evolved from different types of droplet.

In addition, a volume concentration mode was obtained in the sub-micron size with the VMOD of 0.32 ± 0.02 μm. The super-micron SSA were reported to be mainly consisted of Na^+^ and Cl^−^, but the smaller sub-micron particles SSA were mainly consisted of organics and were evolved from film droplets^16^,^17^,^32^. The correlation between 0.3 μm and 2.1 μm modes volume concentrations was significant with r = 0.83, P < 0.01. When we ruled out the three samples collected on 8–10 April 2014, the correlation between 0.3 μm and 2.0 μm modes was poor with r = 0.46, P > 0.05. The large wind speed wind apparently favored the generation of particles in both super- and sub- micrometer, although the super-micron and sub-micron SSA may be evolved from different types of drops.

**Figure 5:**
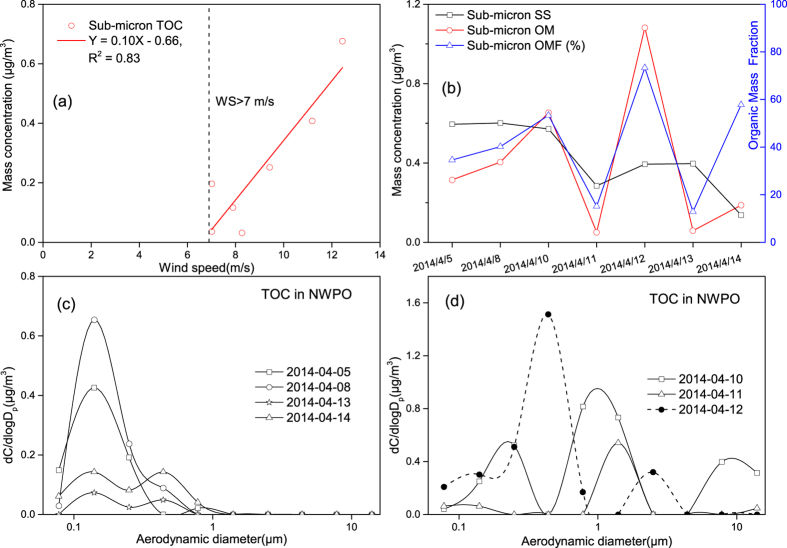
Correlation between TOC and wind speed, OMF and TOC’s size distributions measured in samples collected over the NWPO, (**a**): the linear correlation between wind speed and TOC concentration; (**b**): OMF in sub-micron particles; (**c**,**d**): size distributions of TOC in mass concentration.

To further examine the possibility for wind-speed enhanced sea spray aerosols in sub-micron particles, we plotted the TOC mass concentration in the sub-micron particles against wind speed in different days of Campaign 2. No correlation was obtained when all 13 samples were used, i.e., r = 0.18, P(0.55) > 0.05. For Category-1 samples collected under wind speed >7 m/s, Fig. 5a showed a linear relationship, i.e., [TOC]_sub-micron particles_ = 0.10 × U-0.66, R^2^ = 0.83. TOC in sub-micron particles measured on 12 April 2014 was possibly affected by self-contamination of the vessel on basis of its dominant mode at 0.4 μm, which was also the typical mode size for primary combustion particles^33^. OMF in sub-micron particles ranged from 13% to 58% (Fig. 5b) with the highest on 10 April 2014 when the averaged wind speed was ~11 m/s. As reported by Quinn *et al*.^34^ and review articles^16^,^35^, ocean-derived organics occupied the most mass in SSA with the diameter <0.3 μm and they were negligible in super-micron particles. The measured TOC were increased in <0.3 μm particles, but the super-micron TOC was generally blow the detection limit except three samples mentioned earlier in this study (Fig. 5c,d). The enhanced 0.32 ± 0.02 μm mode in volume concentration under large wind speeds were likely due to more ocean-derived primary organic aerosols generated. The sea surface microlayer (SSML) has physicochemical and biological properties to be measurably distinct from underlying waters and has a potential role in organic enrichment of marine aerosols. As reviewed by Cunliffe *et al*.^36^, SSML can be simply explained as the air-entrainment bubbles rises to sea surface and leads to the accumulation of organics therein, where the organics are scavenged by wind-driven bubble bursting^36^. Although the composition of organics in the SSML varies with microbial community, Quinn *et al*.^34^ suggest that the large reservoir of organic carbon in surface sea water is responsible for the organic carbon enrichment of nascent marine aerosols^34^.

**Figure 6:**
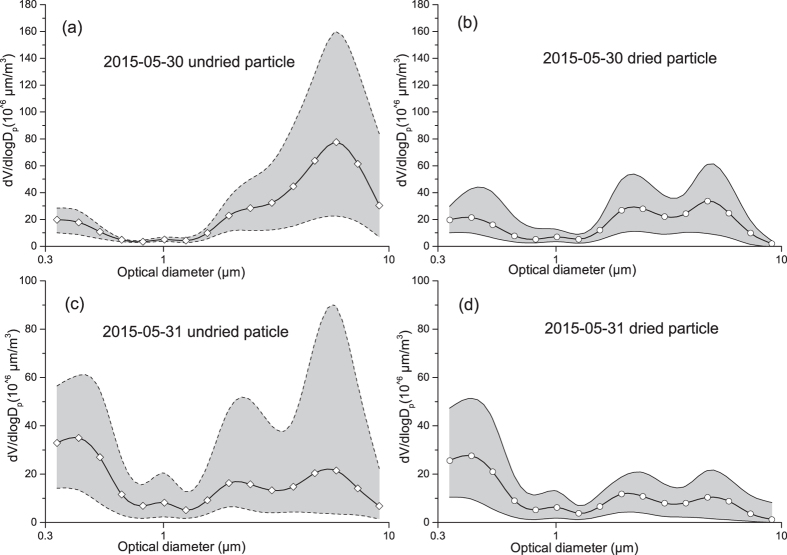
Differences between undried and dried particles measured by OPS, (**a**,**b**): the size distribution of undried/dried particles in 2015-05-30 samples, (**c**,**d**): the size distribution of undried/dried particles in 2015-05-31 samples; A dryer was added for 20–30 minutes alternatively to control wet/dry inlet. The circles represent the mean values of particle volume concentrations, and the solid represents the range (Max and Min) of particle volume concentrations for hundreds of samples on that day as a concise view.

In addition, we used the OPS to make a special measurement on 30–31 May 2015 at the beach site and the tide-effect on the generation of SSA can be thereby investigated. A dryer was installed upstream of the OPS and switched on or off for every 20–30 minutes, which aimed at measuring the different percentage of water content in the two super-micron mode SSA (Fig. 6a–d). The two super-micron modes with the VMOD (dried/undried) of 2.0/2.1 μm and 5.2/5.7 μm were also observed and the 5.2/5.7 μm mode dominated on 31 May 2015. The two modes were consistent with the calculated modes of Na^+^ and those observed in the marine atmospheres.

**Figure 7:**
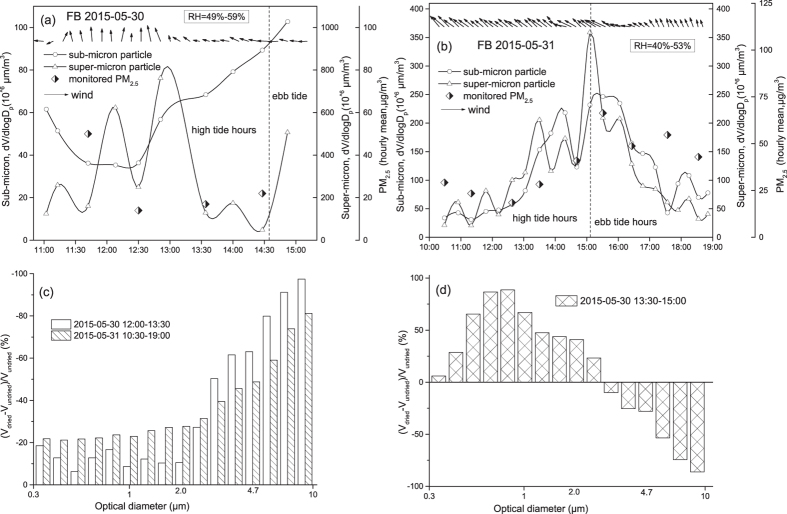
The volume concentrations of sub- and super-micron particles in relation to tide, wind and PM_2.5_, (**a**): the 2015-05-30 samples, under onshore wind during 11:00–13:00 while offshore wind during 13:00–15:00; (**b**): the 2015-05-31 samples, during 10:00–19:00 under dominant onshore wind. (**c**,**d**): the percentages of (V_dried_ − V_undried_)/V_undried_.

On 31 May 2015, the onshore wind was dominated during the sampling period. The increase and decrease of the calculated volume concentrations for super-micron particles perfectly matched the high tide hours during 10:30–15:00 and the ebb tide hours during 15:00–19:00, indicating that the temporal variation was caused by the enhanced and reduced amount of freshly generated SSA (Fig. 7b). The temporal variation of sub-micron particles in volume concentration also matched the tide hours well on the day, but we cannot completely attribute this to varying generation of sea sprayed aerosols. The concentrations of PM_2.5_ measured at ~2 km away from the beach site also increased during the high tide period and decreased during the ebb tide period (Fig. 7b). Anthropogenic aerosols may contribute to the calculated volume concentrations of sub-micron particle. Note that the oscillation of the calculated volume concentrations in every 20–30 minutes was mainly due to switching on or off the dryer. On 30 May, the onshore wind was blowing only at 11:30–13:30. The results observed at the two hours were consistent with those obtained on 31 May (Fig. 7a) and were not detailed.

When the volume concentrations measured without (V_undried_) and with (V_dried_) the dryer were used to calculate the percentage of (V_dried_ − V_undried_)/V_undried_ (Fig. 7c,d), the negative percentage for >2.5 μm atmospheric particles were substantially increased from −81% at 10 μm to −31% at 2.4 μm. However, the decrease for <2.5 μm atmospheric particles narrowly varied from −21% to −28%. The >2.5 μm atmospheric particles apparently contained more water than <2.5 μm atmospheric particles. The increasing percentages with decreasing particle size implied that the larger super-micron mode contained excessive water. The larger super-micron atmospheric particles could be very freshly generated and didn’t achieve an equilibrium with ambient water vapor. During 13:30–15:00 on 30 May, the offshore wind was blowing. Although the negative percentages of (V_dried_ − V_undried_)/V_undried_ were increased from −86% at 10 μm to–10% at 3.0 μm, the positive percentages occurred for the particles less than 2.4 μm. The positive percentage values likely reflected temporal variations of non-SSA instead of SSA.

More OPS data at the beach site were available when no dryer was installed upstream. For example, two super-micron modes of atmospheric particles were also observed on 19–20 January 2015 (Fig. S7). On 23–25 May 2014, the sampling sites were situated at ~50 m away from the intertidal zone and the observed volume concentrations were comparable to those measured in marine atmospheres (Fig. S8).

### Chloride depletion in SSA particles

During Campaign 1, chloride depletion was generally present in atmospheric particles with the aerodynamic diameter <10 μm collected over the open oceans. When the percentages of chloride depletion were averaged over all NWPO samples at each super-micrometer size bin, the average value increased from 8% to 35% with decreasing size from 10 μm to 1 μm (Fig. S9). The size dependence of chloride depletion has been reported in literature^8^,^9^,^10^. Since the equivalent ratios of ([Cl^−^] + [NO_3_^−^]])/[Na^+^] in the super-micron particles were almost equal to the value of 1.16 ([Cl^−^]/[Na^+^] in seawater), the formation of NO_3_^−^ accounted for almost all chloride depletion. Similar results were obtained in atmospheric particles collected over the open oceans during Campaign 2. The size dependence of chloride depletion was also present in the RNWPO samples collected during two campaigns. However, the equivalent ratios of ([Cl^-^] + [NO_3_^−^]])/[Na^+^] in the super-micron particles were larger than 1.16 in the NWPO samples. Nitrate has been found to be associated with soil-derived calcium and ammonium^37^,^38^ and this could be partially true on the RNWPO samples. Moreover, we didn’t find chloride depletion unexplained by NO_3_^−^ and SO_4_^2−^ during Campaign 1 and Campaign 2.

During Campaigns 3–4, only 12 out of the total 29 samples had the ratios of Mg^2+^/Na^+^ close to the value in the seawater (0.11). However, the ratios of Mg^2+^/Na^+^ were close to 0.11 in 24 out of the total 27 samples collected during Campaigns 1–2. The larger ratios of Mg^2+^/Na^+^ implied a notable contribution of continent-derived dust or soil aerosols to super-micron atmospheric particles, which further complicated the calculated chloride depletion. We thereby didn’t analyze chloride depletion during Campaigns 3–4.

For samples collected on 15–16 September at the beach site, the chloride depletion in percentage ranged from 10% to 81% in super-micron atmospheric particles. However, the percentage in the 5.0 μm mode particles were almost same in the two days, i.e., 44 ± 4% in 1.8–10 μm particles on 15 September and 40 ± 4% in the same size particles on 16 September. The percentages in the 1.0–1.8 μm particles were 82% and 53% on 15 and 16 September, indicating that chloride depletion in the mode SSA was enhanced. The equivalent ratios of ([Cl^−^] + [NO_3_^−^]])/[Na^+^] were lower than 1.16 (Fig. S9e) and the ratios of ([Cl^−^] + [NO_3_^−^] + [SO_4_^2−^])/([Na^+^] + [NH_4_^+^]) in each super-micron size bin were almost same as the ratios of ([Cl^−^] + [NO_3_^−^]])/[Na^+^] (not shown). Chloride depletion unexplained by NO_3_^−^ and SO_4_^2−^ may be explained by the reactions releasing Cl_2_, HOCl and the replacement by organic acids^14^,^39^,^40^.

The size dependence of chloride depletion on 18 September was different from that on 15–16 September. On that day, the offshore wind dominated and the observed Na^+^ were sharply reduced (Fig. 2g,h). Moreover, a much high concentration of K^+^ was observed, indicating a strong emission of particles from nearby BBQ sources (Fig. S10). The ratios of ([Cl^−^] + [NO_3_^−^]])/[Na^+^] on 18 September were also larger than 1.16 (Fig. S9e). The BBQ emission and soil-derived particles may complicate the results on 18 September.

### Implication and future works

In literature, the generation of SSA was argued to be affected not only by wind speed, also affected by multiple factors such as seawater salinity, SST, precipitation, SSML properties, etc^15^,^17^,^23^,^26^,^28^,^29^,^36^. We found that the vessel-derived wave-breaking can be an important artificial source of SSA and can greatly affect the [M_SSA_]. However, enhanced wave-breaking may also occur under natural conditions from the marginal seas to the beach. This raised the importance to reexamine those equations which were derived from field measurements and were used to estimate the global flux of SSA. Although the naturally enhanced wave-breaking at the beach can be simply ascribed to the friction effect, the mechanism for naturally enhanced wave-breaking in the marginal seas remains unknown. The same was true for the enhanced generation of SSA and this needs future investigation.

Wave breaking can release film drops, jet drops and spume drops^24^,^27^,^41^,^42^. We found that the SSA in the 1–2 μm mode may be derived from daughter droplets of jet/spume drops and this type of SSA greatly decreased from open oceans to coastal beach sites. In open oceans, the frequency of strong winds is generally higher than coastal seas and beach sites. The global warming has already led to more intense hurricanes^43^. Latham & Smith reported the existence of increased emissions of SSA caused by the increasing wind speed which results from atmospheric warming^44^. The high wind speed (>7 m/s) can enhance the generation of the 1–2 μm mode SSA more than the 5.0 μm mode as shown in this study. Thus, global warming may not only generate more SSA, but also change their size distributions as well as their direct and indirect climate feedbacks.

The 1–2 μm mode SSA had a longer residence time in the atmosphere when comparing to the 5.0 μm mode SSA. Therefore, its relative abundance will increase with the distance from the sea surface in horizontal and vertical directions. To improve the accurate of the estimated global SSA flux evolved from drops, a high time-resolution wind speed is required. Moreover, the 1.5 μm mode SSA appeared to play an important role in N cycling in marine atmospheres. For example, the averaged concentration of NO_3_^−^ in 1.5 μm mode particles were even larger than that in 5.0 μm mode particles collected in open oceans during Campaign 2 (Figs S11 and S12).

## Methods

### Marine campaigns

In this study, four cruising campaigns we participated were referring to Campaigns 1–4. In Campaign 1, the research vessel (R/V Dong Fang Hong 2) cruised from the YS to the NWPO during 31 March–4 May 2015. In Campaign 2, the vessel also cruised from the YS to the NWPO during 18 March–15 April 2014. Campaign 3 and Campaign 4 were performed in the YS and the BS during 6–24 November 2013 and 2–19 November 2012, respectively. During these campaigns, a suite of off-line particle samplers such as two types of MOUDI and high-volume TSP and PM_2.5_ samplers, gas analyzers including SO_2_, NO_x_ and O_3_ analyzers (Thermo Fisher Scientific), on-line aerosol instruments such as an optical particulate matter size spectrometer (OPS, TSI Incorporated, 3330), fast mobility particle size spectrometer (FMPS, TSI Incorporated, 3901), Condensation Particle Counter (CPC, TSI Incorporated, 3775), etc., were used to measure atmospheric gases and particles. All facilities were deployed on the vessel’s upper deck and were approximately 8 m above the sea surface. Particle samplers were switched off when the vessel anchored for other surveys. The durations of aerosol particles collected by particle samplers vary from tens to dozens of hours. All gas analyzers and on-line aerosol instruments were operated during the whole campaign period except for the maintenance. Meteorological data was obtained from the Vessel Management System (VMS). The wind velocity was corrected by deducting the influence of vessel sailing and was used for data analysis in this study.

### Coastal observations

To compare with those measurements made in marginal seas and open oceans, we conducted observations at three beach sites of the YS during 24 May 2014, 19–20 January, 30–31 May and 15–18 September 2015 (Fushan rocky beach site, FB), during 25 May 2014 (Luxun park rocky beach site, LP) and during 23–24 May 2014 (Shilaoren sandy beach site, SB). In addition, we also collected atmospheric particles at a coastal semi-urban site located at the campus of the Ocean University of China (OUC) during 20 May-2 June 2013 and 18 Novmber–30 December 2015. The OUC site is ~7 km from the YS.

### Instruments

A nano-MOUDI-II™ (Models 122-NR) was used to collect atmospheric particles in Campaigns 1, 3–4, at a beach site and at the semi-urban coastal site. The MOUDI had 14 size bins with 50% cut-off aerodynamic diameters at 0.01, 0.018, 0.032, 0.056, 0.10, 0.18, 0.32, 0.56, 1.0, 1.8, 3.2, 5.6, 10.0 and 18.0 μm at an airflow rate of 29.4 L/min. It was equipped with Teflon filters (47 mm, Pall Life Sciences) in the upper 11 stages and Zefluor filters in the remaining three stages. A MOUDI (Models110-II™) including 11 size bins from 0.054 to 18.0 μm was used in sampling during Campaign 2. It was equipped with quartz filters (pre-combusted at 600 °C for 6 hours) to collect atmospheric particles. In addition, an OPS including 16 channels at 0.338, 0.421, 0.524, 0.653, 0.812, 1.011, 1.259, 1.568, 1.952, 2.43, 3.026, 3.767, 4.690, 5.839, 7.270, 9.051 μm (optical diameter) was used to simultaneously measure particle number concentrations or to operate alone in different sampling periods. A FMPS was used to measure number concentrations of atmospheric particles from ~5.6 nm to 0.56 μm in mobility diameter.

### Chemical analysis

The particle samples collected on Teflon/quartz filters were wrapped in aluminum foil (prebaked at 450 °C for 4 hours in a furnace to eliminate the absorbed organic compounds), then stored at −20 °C refrigerator in darkness before handle them in a clean lab. The samples were extracted using deionized water and the extracts were then analyzed for Cl^−^, SO_4_^2−^, NO_3_^−^, oxalate, Na^+^, Ca^2+^, Mg^2+^ and NH_4_^+^ etc., by ICS-1100 (Dionex). For samples collected on quartz filters, a half piece of filter was used for extraction. 2–3 field blank samples in each campaign were obtained with those particle samples. Details on chemical analysis and QA/QC methods can be found therein^45^,^46^. In Campaign 2, mass concentration of total organic carbon (TOC) and element carbon (EC) in the collected particles were also determined by the thermal/optical carbon analyzer (Sunset Laboratory Inc., Forest Grove, OR) and the method has been detailed in Feng *et al*.^13^.

### Statistical analysis of the data

In this article, the molar ratios of Cl^−^/Na^+^ and Mg^2+^/Na^+^ in seawater are assumed to be 1.16 and 0.11, respectively^32^. And the seawater ratio of (Na^+^ + Mg^2+^ + Ca^2+^ + K^+^ + Cl^−^ + SO_4_^2−^ + HCO_3_^−^)/Na^+^ is assumed to be 3.26^47^. Assuming that the measured Na^+^ in super-micron particles completely comes from SSA in the atmosphere of open oceans and the beach site, Formula 1 and 2 can be used to calculate the M_SSA_ and chloride depletion (%) therein^9^. Formula 1 and 2 were also used to calculate the sub-micron M_SSA_ in the atmosphere of open oceans, but they were not valid for other marine and coastal atmospheres due to complex sources of Na^+^ in those sub-micron particles. The organic mass fraction (OMF) is calculated by Fomula 3, in which the organic mass was equal to the measured organic carbon multiplied by a factor of 1.6^13^. Note that the factor was chosen as 1.4 −1.6 in literature, depending on organic chemical components which were not available in this study. A multiple log-normal function using Twomey algorithm was adopted to fit the modes of SSA. The size range of SSA where the concentration peaked represents the fitted mode mass of SSA, e.g., M_SSA_ within 1–1.8 μm represents the 1.5 μm mode mass of SSA. Pearson correlation coefficient (r) was adopted for correlation analysis. Determination coefficient (R^2^) was adopted for function fitting.


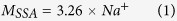



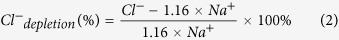



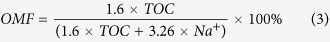


## Additional Information

**How to cite this article**: Feng, L. *et al*. Insight into Generation and Evolution of Sea-Salt Aerosols from Field Measurements in Diversified Marine and Coastal Atmospheres. *Sci. Rep.* **7**, 41260; doi: 10.1038/srep41260 (2017).

**Publisher's note:** Springer Nature remains neutral with regard to jurisdictional claims in published maps and institutional affiliations.

## Supplementary Material

Supplementary Information
