# Supplementary material for: Insight into Generation and Evolution of Sea-Salt Aerosols from Field Measurements in Diversified Marine and Coastal Atmospheres
Source: Sci Rep. 2017 Jan 25;7:41260. doi: 10.1038/srep41260 (PMC5264635; doi:10.1038/srep41260)

Supplementary Information of

Insight into generation and evolution of sea-salt aerosols from field measurements in diversified marine and coastal atmospheres

Limin Feng [1], Hengqing Shen[2], Yujiao Zhu[1], Huiwang Gao[1,3], Xiaohong Yao[1,3*]

[1]Key Lab of Marine Environmental Science and Ecology, Ministry of Education, Ocean University of China, Qingdao, China

[2]State Key Laboratory of Environmental Simulation and Pollution Control, College of Environmental Sciences and Engineering, Peking University, Beijing 100871, China

[3]Qingdao Collaborative Center of Marine Science and Technology, Qingdao 266100, China

*Corresponding authors: Xiaohong Yao, email: xhyao@ouc.edu.cn

Table of contents:

Type	Number
Table	**1**
Figures	**12**

Table S1 The mass concentrations of element carbon (EC, μg/m^3) in Campaign 2 (NWPO, 2014).

Size \ Sample	Mar. 19	Mar. 21	Mar. 25	Apr. 12
0.078 μm	0.00	0.16	0.01	0.00
0.14 μm	0.00	0.61	0.02	0.00
0.25 μm	0.00	0.41	0.17	0.02
0.44 μm	0.00	0.23	0.00	0.27
0.78 μm	0.03	0.24	0.00	0.00
1.4 μm	0.00	0.02	0.00	0.00
2.5 μm	0.00	0.00	0.00	0.00
4.4 μm	0.00	0.00	0.00	0.00
7.8 μm	0.00	0.00	0.00	0.00
14 μm	0.00	0.00	0.00	0.00
No detected	Apr. 5, Apr. 6, Apr. 8, Apr. 10, Apr. 11, Apr.13, Apr. 14, Apr.15.			

Fig. S1 The foams caused by Dong Fang Hong 2 vessel breaking wave under the calm wind condition in the Bohai Sea. This photo was taken by Limin Feng using his mobile phone. He agrees to submit this photo in the Scientific Report for an *Open Access* publication in any form.

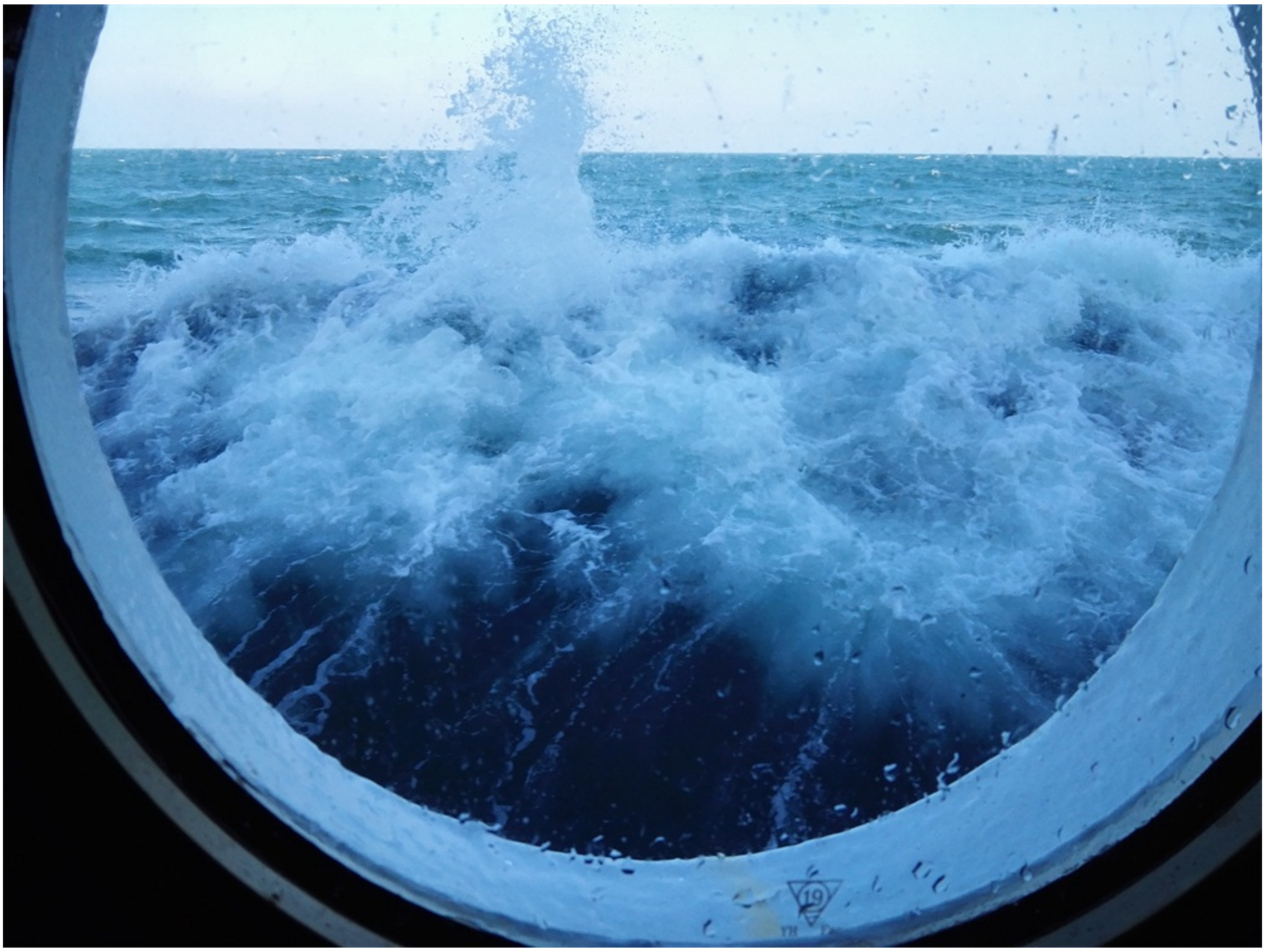

Fig. S2 The estimated M_{SSA} values in super-micron particles against the wind speed in Campaign 3-4.

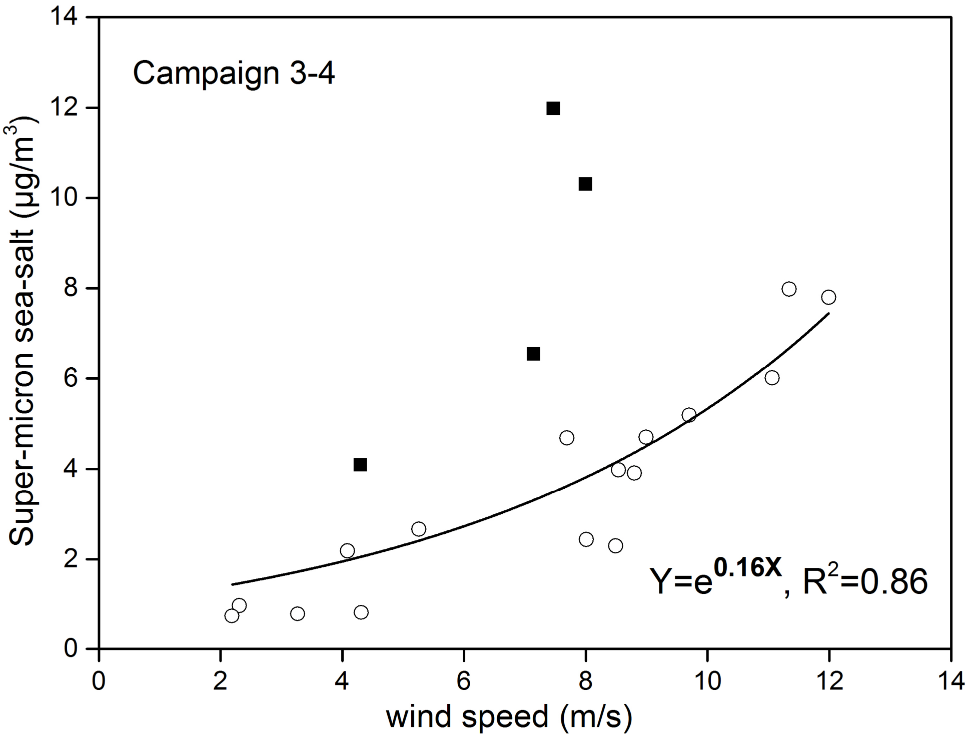

Fig. S3 Continuous log-normal size distribution and modal fitting of sea-salt ions calculated by the Twomey algorithm in Campaign 1-2.

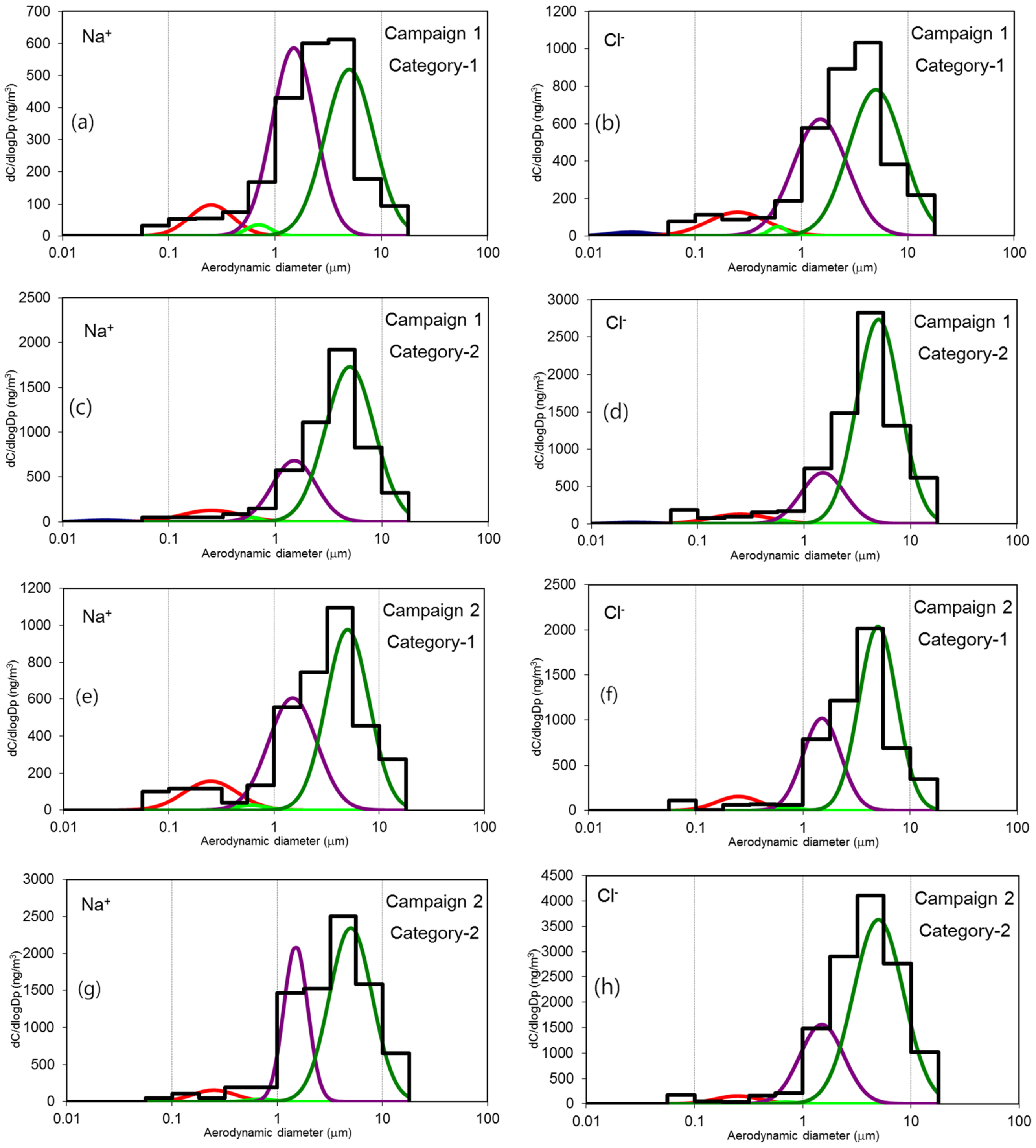

Fig. S4 Continuous log-normal size distribution and modal fitting of sea-salt ions calculated by the Twomey algorithm in samples collected at the OUC site.

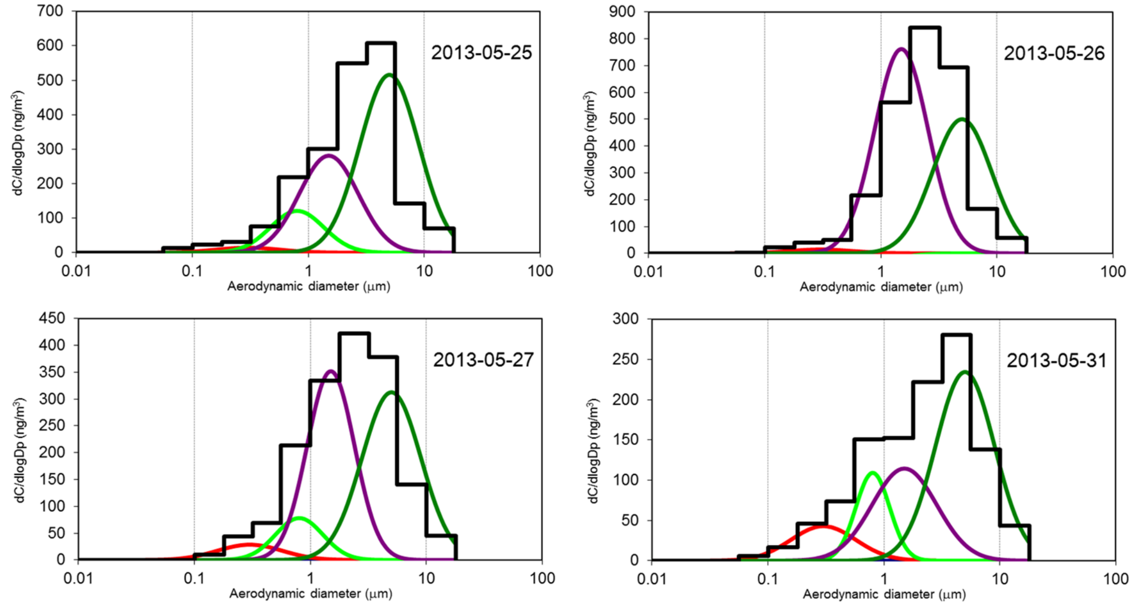

Fig. S5 Size distributions of K^+ and Cl^- in the OUC site.

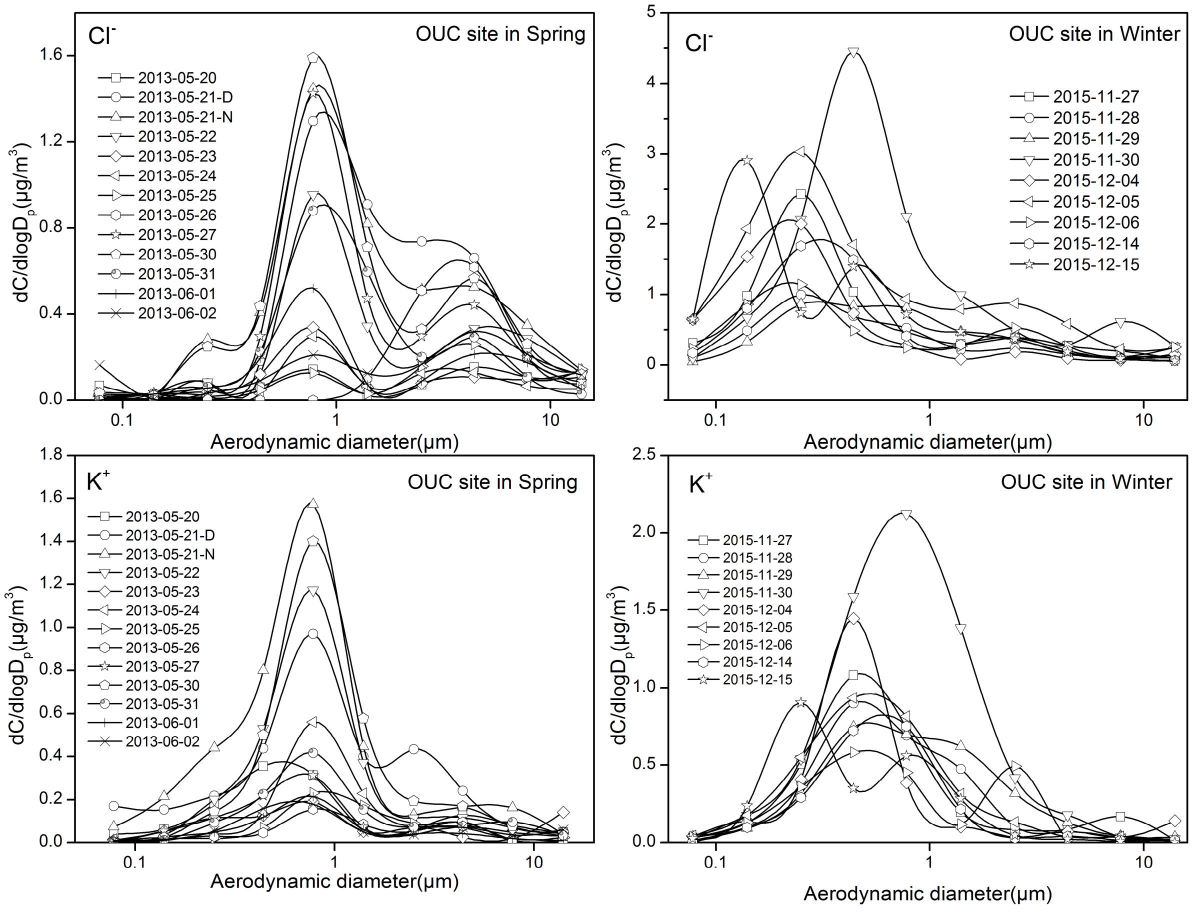

Fig. S6 Number concentration of particles measured by OPS and FMPS in the NWPO, 2014.

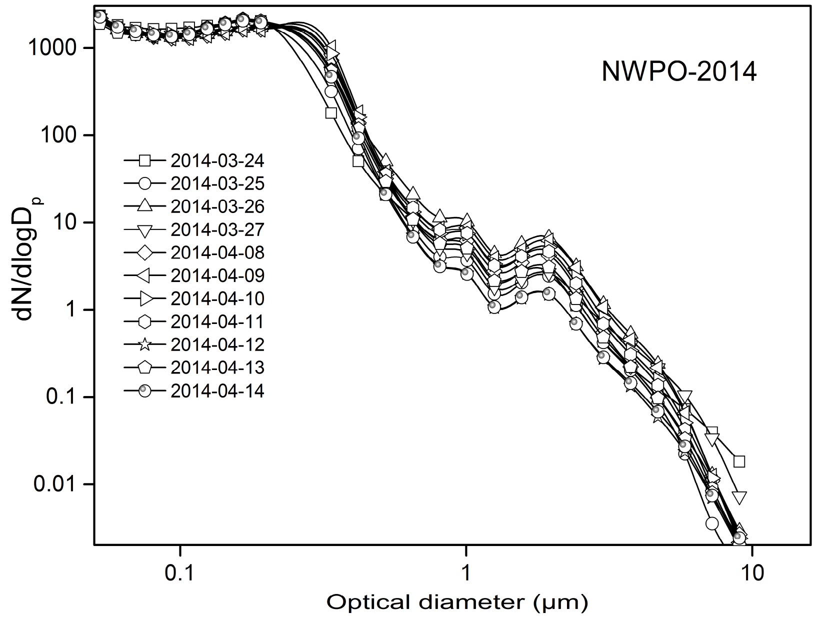

Fig. S7 Online particle samples collected by OPS in the Fushan beach. The 2015-01-19 (dominant offshore wind) and 2015-01-20 (dominant onshore wind) was sampling without a dryer.

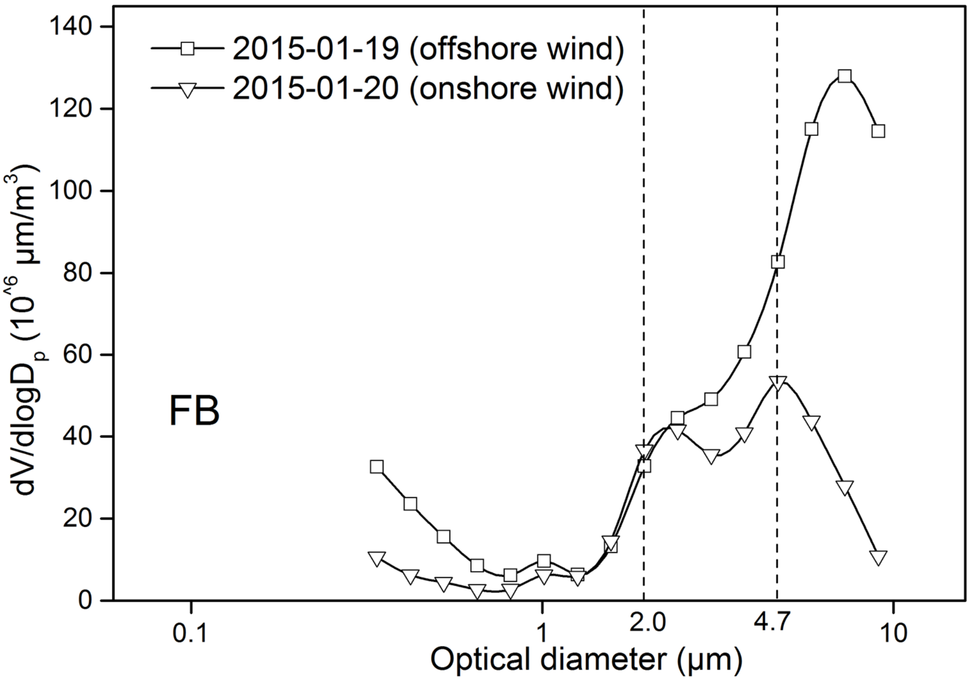

Fig. S8 Online particle samples collected by OPS in Fushan beach (rocky beach, solid symbol), Luxun Park (rocky beach, solid line) and Shilaoren beach (sandy beach, dash line).

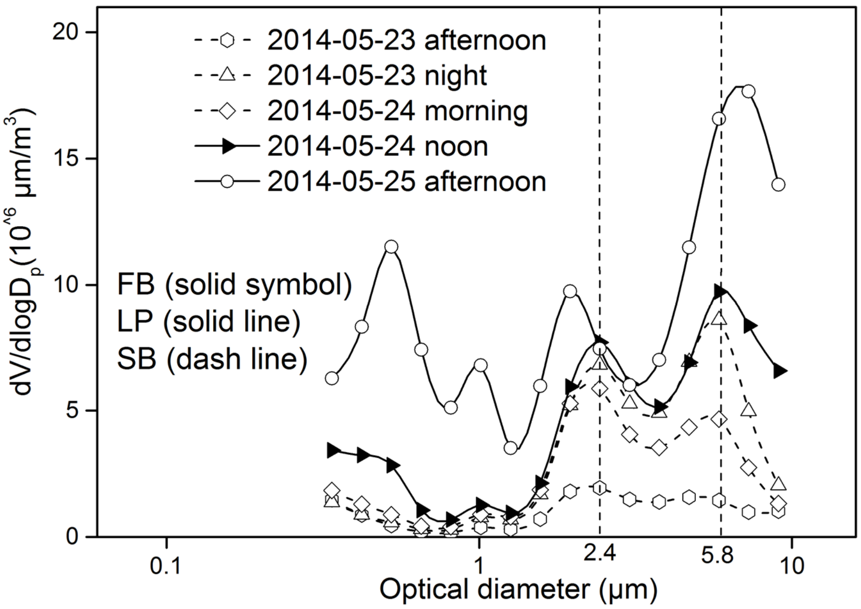

Fig. S9 Chloride depletion and contribution of inorganic acids, (a): NWPO-2015 samples in campaign 1; (b): roundtrips samples in Campaign 1; (c): NWPO-2014 samples in Campaign 2; (d): roundtrip samples in Campaign 2; (e): Fushan beach samples, dash lines for episode sample due to human being contribution.

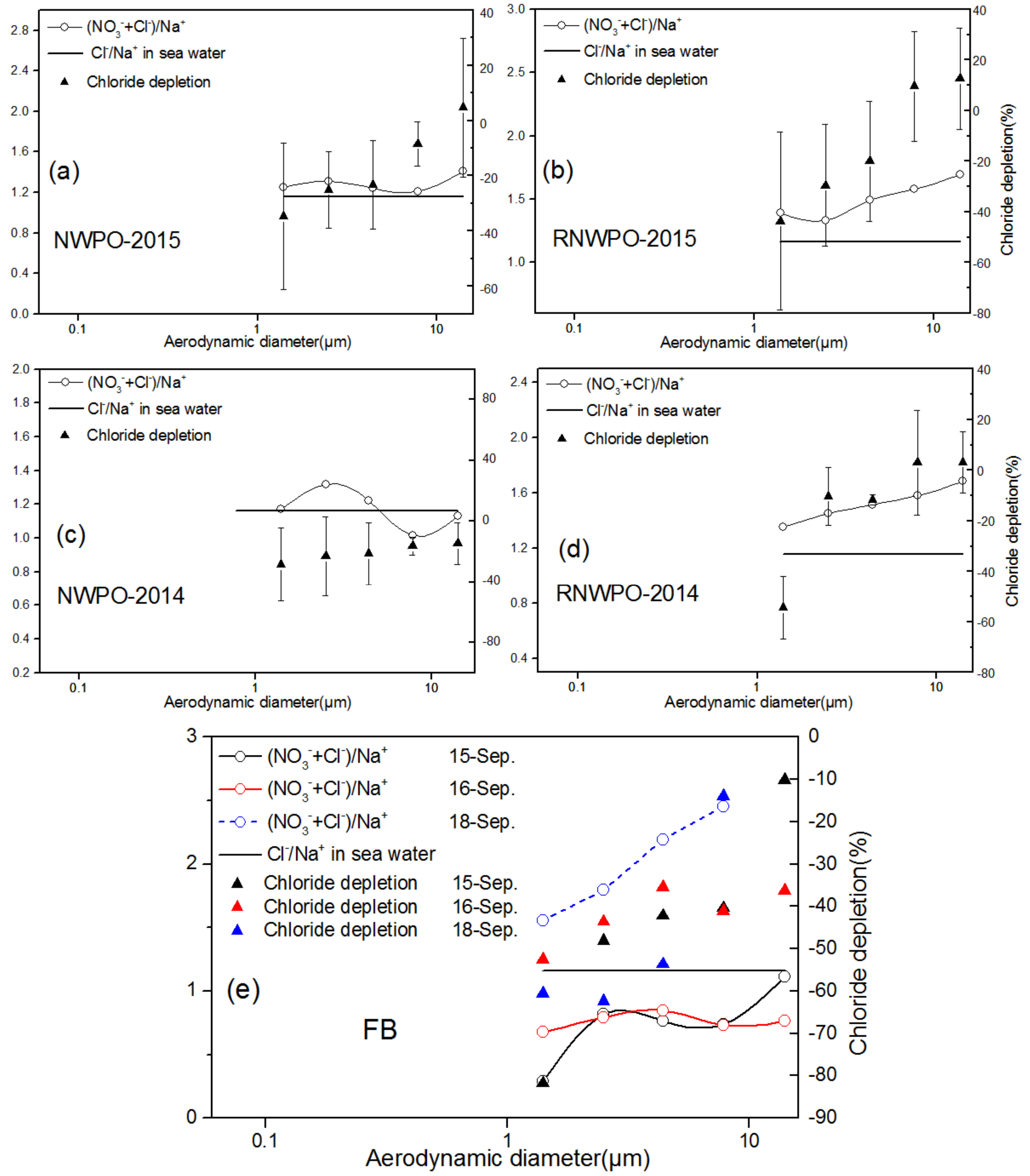

Fig. S10 The size distributions of K^+, Mg^{2+}, Ca^{2+}, SO_4^{2-}, NO_3^-, Cl^-, and NH_4^+ in Fushan beach samples. The 2015-09-18 samples appeared significant human-being contribution.

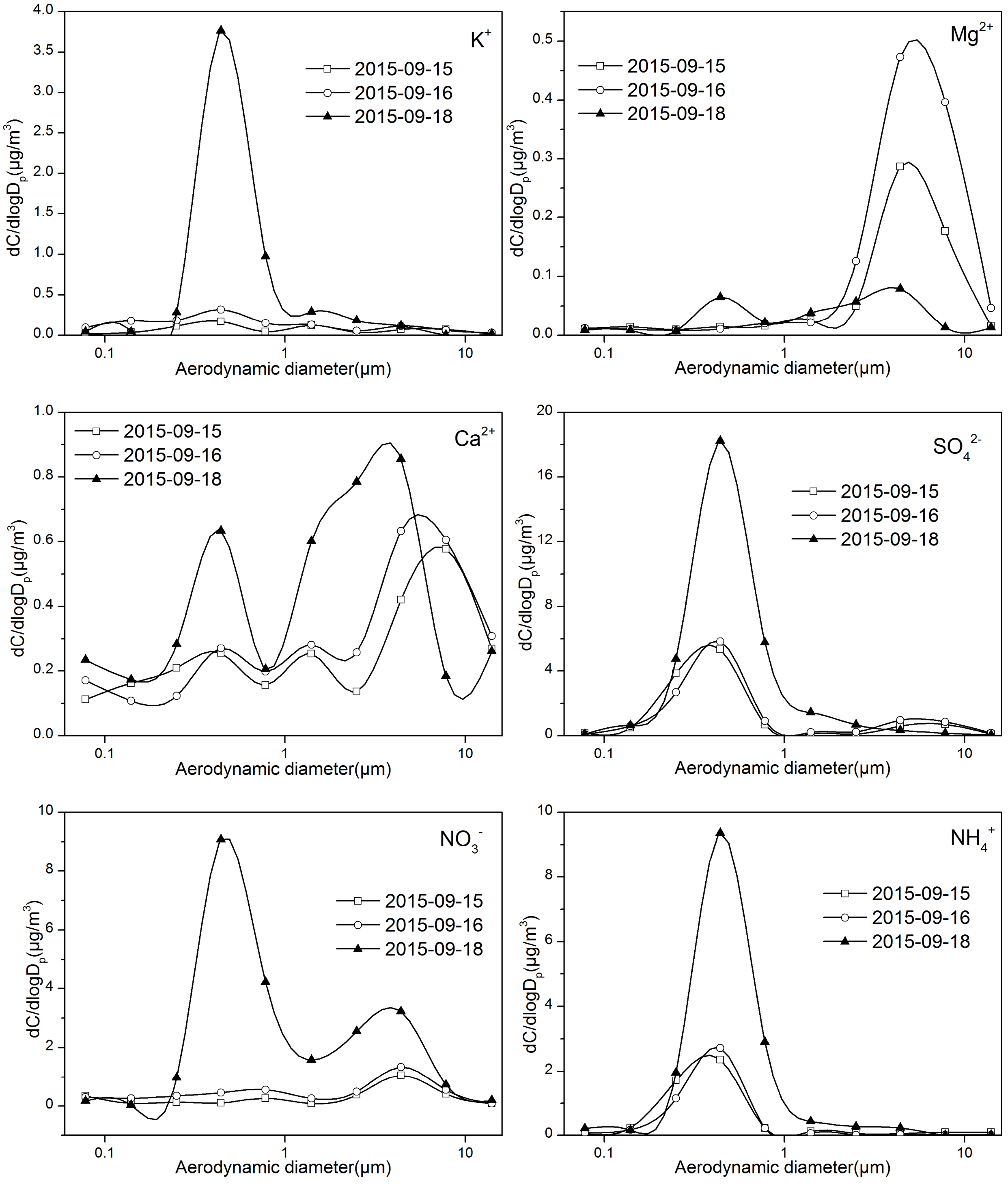

Fig. S11 The size distribution of NO_3^- in Campaign 2.

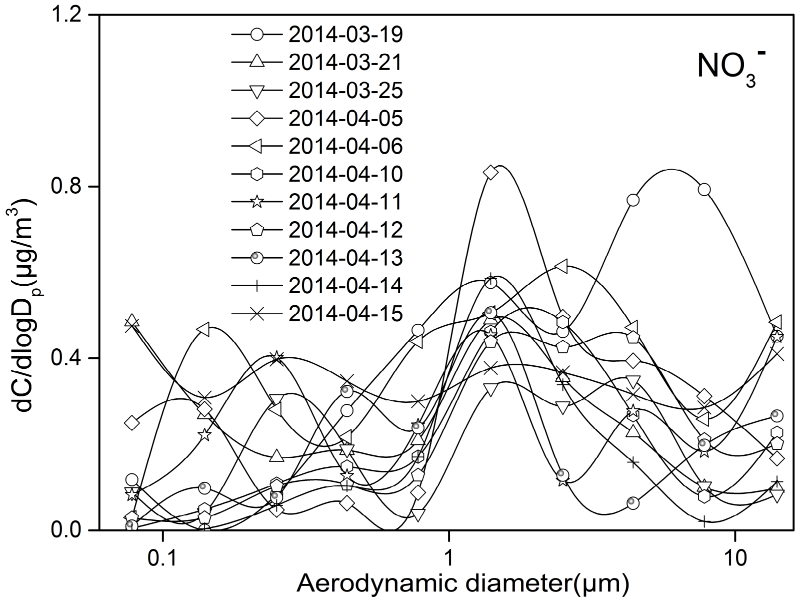

Fig. S12 The size distributions of nss-SO_4^{2-}, Na^+, NO_3^-, Cl^-, and NH_4^+ in the NWPO and roundtrips samples of Campaign 1-2.

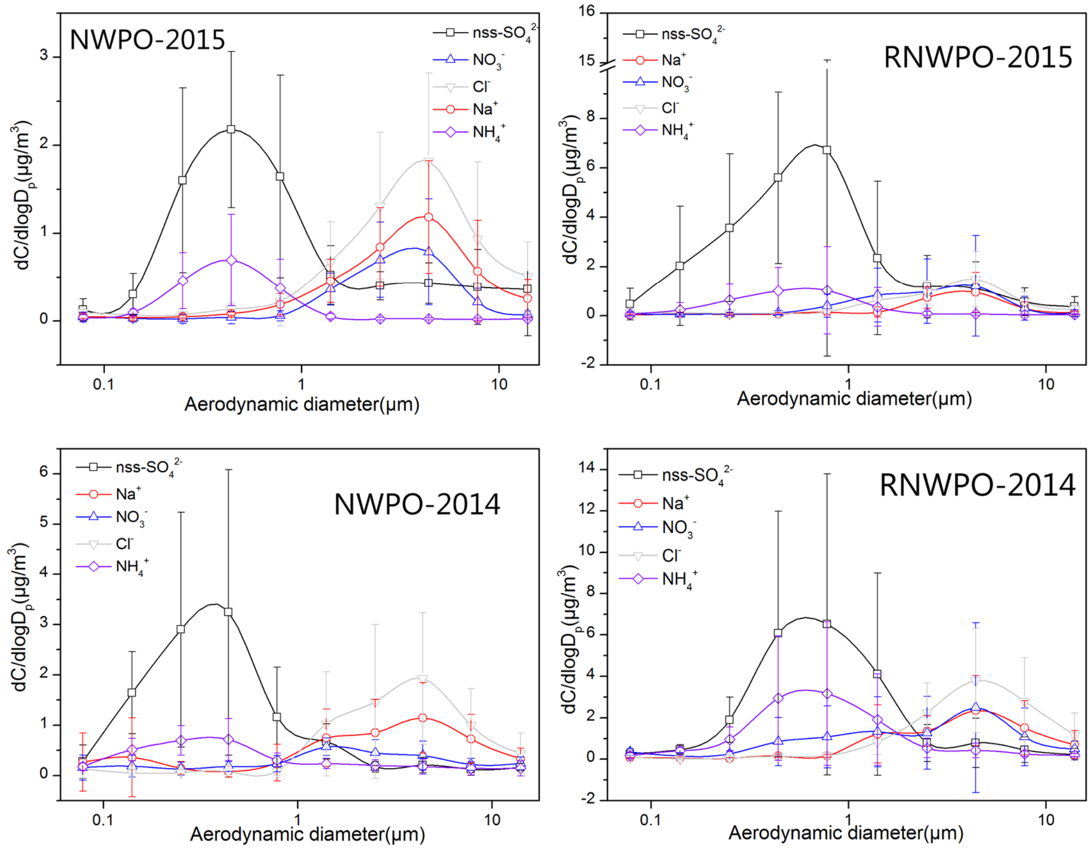

Supplement: Supplementary Information [file srep41260-s1.pdf]
